# Draft Genome Sequence of *Bacillus coagulans* GBI-30, 6086, a Widely Used Spore-Forming Probiotic Strain

**DOI:** 10.1128/genomeA.01080-14

**Published:** 2014-11-06

**Authors:** Luigi Orrù, Elisa Salvetti, Luigi Cattivelli, Antonella Lamontanara, Vania Michelotti, Vittorio Capozzi, Giuseppe Spano, David Keller, Howard Cash, Alessia Martina, Sandra Torriani, Giovanna E. Felis

**Affiliations:** a Consiglio per la Ricerca e la Sperimentazione in Agricoltura, Genomics Research Centre, Fiorenzuola d’Arda, Piacenza, Italy; b Department of Biotechnology, University of Verona, Verona, Italy; c Department of Agriculture, Food and Environment Sciences, University of Foggia, Foggia, Italy; d Ganeden Biotech Inc., Mayfield Heights, Ohio, USA

## Abstract

*Bacillus coagulans* GBI-30, 6086 is a safe strain, already available on the market, and characterized by certified beneficial effects. The draft genome sequence presented here constitutes the first pillar toward the identification of the molecular mechanisms responsible for its positive features and safety.

## GENOME ANNOUNCEMENT

*Bacillus coagulans* strain GBI-30, 6086 is a safe, spore-forming strain, as testified by the GRAS (Generally Recognized As Safe) status received in 2012 from the United States FDA; the strain is authorized for human consumption and already available in a wide selection of functional foods or as dietary supplement (1). This strain is characterized by certified beneficial effects in gastrointestinal disorders, such as irritable bowel syndrome, intestinal gas, and colitis (2, 3, 4, 5); in rheumatoid arthritis (6); and in common viral infections of the respiratory tract (7). As a sporeformer, *B. coagulans* GBI-30, 6086 can be incorporated into foods, where it can survive the mild heat-treatments used for sterilization andwithstand the harsh conditions of the gastrointestinal tract, i.e., the low pH of the gastric barrier (8).

Here we report the draft genome sequence of *B. coagulans* GBI-30, 6086 in order to unveil the genetic basis of its safety and probiosis. To the best of our knowledge, this is the first published fully assembled genome of a commercial *B. coagulans* probiotic strain.

The whole-genome sequencing was performed using the Illumina GAIIx platform at CRA-Genomics Research Centre (Piacenza, Italy) with a paired-end library; the reads were *de novo* assembled using the CLC Genomic Workbench version 7.0. The genome sequence was annotated by the NCBI Prokaryotic Genomes Annotation Pipeline.

A total of 14,500,000 paired-end reads of 110-bp length on average (genome coverage of 840×) were assembled into 224 contigs (*N*_50_ length of 44,706 bp), with the largest assembled contig of 125,999-bp length. The draft genome consists of 3,458,655 bp with GC % content of 46.38.

A total of 3,373 genes were predicted, of which 3,197 are coding sequences (CDS), 18 are rRNAs, and 82 are tRNAs; 79 were identified to be pseudogenes, and 1 was identified as ncRNA. The genome also contains 3 CRISPR arrays, which could be involved as a defense mechanism toward foreign genetic elements (9).

The strain is predicted to encode for about 500 proteins involved in central carbohydrate metabolism, including glycolysis, pentose phosphate, and xylose utilization pathways (9, 10). As expected, about 80 genes regarding dormancy and sporulation were annotated, in particular, spore DNA protection, sporulation mechanisms, spore core dehydration, and spore germination. Furthermore, the genome contains determinants involved in the adhesion (i.e., fibronectin- and mucus-binding proteins) and active metabolism in the host (as a biotin biosynthesis pathway).

The complete genome of *B. coagulans* GBI-30, 6086 obtained in the present study will contribute to a wider and deeper insight into the safety features of this strain, and the comparative genomic analysis with other *Bacillus* strains genomes (11, 12, 13) might shed new light on the molecular mechanisms at the basis of its probiotic and beneficial properties.

### Nucleotide sequence accession numbers.

This whole-genome shotgun project has been deposited in DDBJ/EMBL/GenBank under the accession number JPSK00000000. The version described in this paper is the first version, JPSK01000000.

## References

[1] Cutting SM 2011 *Bacillus* probiotics. Food Microbiol. 28:214–220. 10.1016/j.fm.2010.03.007. 21315976

[2] Dolin BJ 2009 Effects of a proprietary *Bacillus coagulans* preparation on symptoms of diarrhea-predominant irritable bowel syndrome. Methods Find. Exp. Clin. Pharmacol. 31:655–659. 10.1358/mf.2009.31.10.1441078. 20140275

[3] Hun L 2009 *Bacillus coagulans* significantly improved abdominal pain and bloating in patients with IBS. Postgrad. Med. 121:119–124. 10.3810/pgm.2009.03.1984. 19332970

[4] Kalman DS Schwartz HI Alvarez P Feldman S Pezzullo JC Krieger DR 2009 A prospective, randomized, double-blind, placebo-controlled parallel-group dual site trial to evaluate the effects of a *Bacillus coagulans-*based product on functional intestinal gas symptoms. BMC Gastroenterol. 9:85. 10.1186/1471-230X-9-85. 19922649 PMC2784472

[5] Fitzpatrick LR Small JS Greene WH Karpa KD Farmer S Keller D 2012 *Bacillus coagulans* GBI-30, 6086 limits the recurrence of *Clostridium difficile*-induced colitis following vancomycin withdrawal in mice. Gut Pathog. 4:13 http://www.gutpathogens.com/content/4/1/13. 23088680 10.1186/1757-4749-4-13 PMC3484022

[6] Mandel DR Eichas K Holmes J 2010 *Bacillus coagulans*: a viable adjunct therapy for relieving symptoms of rheumatoid arthritis according to a randomized, controlled trial. BMC Complement. Altern. Med. 10:1. 10.1186/1472-6882-10-1. 20067641 PMC2826289

[7] Jurenka JS 2012 Bacillus coagulans. Altern. Med. Rev. 17:76–81. 22502625

[8] Barbosa TM Serra CR La Ragione RM Woodward MJ Henriques AO 2005 Screening for *Bacillus* isolates in the broiler gastrointestinal tract. Appl. Environ. Microbiol. 71:968–978. 10.1128/AEM.71.2.968-978.2005. 15691955 PMC546680

[9] Su F Tao F Tang H Xu P 2012 Genome sequence of the thermophile *Bacillus coagulans* hammer, the type strain of the species. J. Bacteriol. 194:6294–6295. 10.1128/JB.01380-12. 23105047 PMC3486356

[10] Su F Xu P 2014 Genomic analysis of thermophilic *Bacillus coagulans* strains: efficient producers for platform bio-chemicals. Scientific Rep. 4:1–10. 10.1038/srep03926. PMC3905273 24473268

[11] Rhee MS Moritz BE Xie G del Rio TG Dalin E Tice H Bruce D Goodwin L Chertkov O Brettin T Han C Detter C Pitluck S Land ML Patel M Ou M Harbrucker R Ingrram LO Shanmugam KT 2011 Complete genome sequence of a thermotolerant sporogenic lactic acid bacterium, *Bacillus coagulans* strain 36D1. Stand Genomics Sci. 5:331–340. 10.4056/sigs.2365342. PMC3368420 22675583

[12] Su F Xu K Zhao B Tai C Tao F Tang H Xu P 2011 Genome sequence of the thermophilic strain *Bacillus coagulans* XZL4, an efficient pentose-utilizing producer of chemicals. J. Bacteriol. 193:6398–6399. 10.1128/JB.06157-11. 22038963 PMC3209230

[13] Xu K Su F Tao F Li C Ni J Xu P 2013 Genome sequences of two morphologically distinct and thermophilic *Bacillus coagulans* strains, H-1 and XZL9. Genome Announc. 1(3):e00254-13. 10.1128/genomeA.00254-13. 23682151 PMC3656213

